# The effect of community-based support for caregivers on the risk of virological failure in children and adolescents with HIV in Harare, Zimbabwe (ZENITH): an open-label, randomised controlled trial

**DOI:** 10.1016/S2352-4642(17)30051-2

**Published:** 2017-11

**Authors:** Rashida A Ferrand, Victoria Simms, Ethel Dauya, Tsitsi Bandason, Grace Mchugh, Hilda Mujuru, Prosper Chonzi, Joanna Busza, Katharina Kranzer, Shungu Munyati, Helen A Weiss, Richard J Hayes

**Affiliations:** a Clinical Research Department, London School of Hygiene & Tropical Medicine, London, UK; b MRC Tropical Epidemiology Group, Department of Infectious Disease Epidemiology, London School of Hygiene & Tropical Medicine, London, UK; c Department of Population Health, London School of Hygiene & Tropical Medicine, London, UK; d Biomedical Research and Training Institute, Harare, Zimbabwe; e Department of Paediatrics, University of Zimbabwe, Harare, Zimbabwe; f Harare City Health Department, Harare, Zimbabwe; g National Reference Laboratory, Research Centre Borstel, Germany

## Abstract

**Background:**

Children and adolescents have poorer HIV treatment outcomes than adults. We aimed to assess the effect of community-based support for caregivers of HIV-infected children and adolescents, who are key mediators to children engaging with care, on treatment outcomes.

**Methods:**

In this open-label, randomised contolled trial, we recruited children aged 6–15 years with newly-diagnosed HIV attending primary health-care clinics in Harare, Zimbabwe. Children were randomly assigned to receive decentralised primary health-care clinic-based HIV care (control group), according to national guidelines for 18 months, or decentralised care plus structured support visits by trained community health workers (intervention group) according to national guidelines for 18 months. Primary outcomes were the proportion of participants who died or had an HIV viral load of 400 copies per mL or higher at 12 months after antiretroviral therapy (ART) initiation (among those who started ART within 6 months of enrolment); and the proportion who missed two or more scheduled clinic visits by 18 months post-enrolment (among all participants). Analyses were complete-case, modified-intention-to-treat. This trial is registered with the Pan African Clinical Trials Registry, number PACTR201212000442288.

**Findings:**

Between January, 2013, and January, 2015, 470 participants tested HIV-positive at seven study primary health-care clinics and were screened for eligibility. Of the 334 eligible children and adolescents, 166 were randomly assigned to the intervention group and 168 to the control group. The median age of participants was 11 years (IQR 8–13) and 178 (53%) were girls. Among the 238 participants who started ART within 6 months of enrolment, the proportion who died or had a viral load of 400 copies/mL or higher at 12 months post-ART initiation was significantly lower in the intervention group than in the control group (31 [33%] of 94 participants *vs* 42 [49%] of 86 participants, respectively, adjusted odds ratio [aOR] 0·46, 95% CI 0·23–0·89; p=0·02). The proportion of children missing two or more scheduled visits was similar in the intervention group and control group (27 [17%] of 155 *vs* 30 [18%] of 165, aOR 0·92, 95% CI 0·49–1·74; p=0·79). One participant withdrew from the trial 240 days after enrolment and 12 died during follow-up (five in the intervention group; seven in the control group).

**Interpretation:**

Community-based support for caregivers has high potential for scalability and could have a substantial effect on HIV virological suppression in children and adolescents, a group with disproportionately poor treatment outcomes.

**Funding:**

Wellcome Trust.

## Introduction

The scale-up of interventions to prevent mother-to-child HIV transmission has led to a halving in HIV infections in children since 2000.^1^ However, the number of HIV-related deaths among the generation of children who are surviving to adolescence is rising despite the expansion of antiretroviral therapy (ART) coverage worldwide.^2^ This trend is being driven mainly by mortality in sub-Saharan Africa, where 90% of the world's HIV-infected children live.^3^ Multiple factors drive this mortality, but most paths eventually lead to poor ART adherence and problems with retention in HIV care. Findings of studies in both high-income and low-income settings have shown poorer outcomes in children and adolescents compared with adults across the HIV care cascade, with the proportion virally suppressed as low as 63% at 12 months after ART initiation in those younger than 18 years, and consequent high risk of treatment failure and mortality.^3^, ^4^ However, few interventions have focused on improving HIV-related outcomes in this age group.^5^, ^6^

The pressure on health systems in high HIV prevalence resource-limited settings has led to the decentralisation of HIV services from secondary to primary care and task-shifting to nurses and non-clinical staff.^7^ These strategies, although having shown equivalent outcomes, are reaching their limits as increasing numbers of patients initiate ART, overwhelming workforce capacity.^8^, ^9^ Community based interventions to support HIV care outside clinics are being increasingly promoted to relieve pressure on health facilities and provide more patient-centred services. A systematic review of community-based interventions to support adherence to treatment and retention in care noted similar outcomes to facility-based ART delivery.^10^ However, the interventions were mainly implemented among clinically stable adult patients; only three of the 22 studies included children. As ART is scaled up, community-based approaches could be a mechanism for meeting the growing health needs of children and adolescents in high HIV prevalence settings.^8^

**Research in context**

**Evidence before this study**

Maintaining HIV viral suppression is essential to reduce the risk of mortality, onward transmission of HIV, and development of drug resistance. This requires an individual who is diagnosed with HIV to link to care, start antiretroviral therapy (ART), remain in care, and have sustained high levels of treatment adherence. We searched Medline for systematic reviews of any interventions to improve either linkage, adherence, or retention in care up to February, 2017, using the search terms *HIV or Antiretroviral therapy or ART or ARV) AND (adherence) AND (review or meta-analysis). We identified three systematic reviews investigating interventions aimed at improving viral suppression in HIV-infected individuals receiving ART, and two reviews focused on improving treatment outcomes in HIV-infected adolescents. A meta-analysis included 47 trials reporting virological outcomes, of which three were done in youth or children, two included adolescents (although age-disaggregated data were not presented), and the remainder were done in adults. Of the 16 intervention packages, cognitive behaviour therapy (OR 1·46, 95% CI 1·05–2·12) and supporter interventions (1·28, 1·01–1·71) were superior to standard of care. Only 12 of the trials were done in low and middle-income countries (LMICs) and none showed an effect of interventions on viral load outcomes. Similarly, systematic reviews investigating the effect of peer interventions (17 trials of which eight were from LMICs, and one included children and adolescents) reported no effect on viral suppression; and home-based interventions among HIV infected adults in sub-Saharan Africa reported only a small and non-significant effect (pooled OR 1·13, 95% CI 0·51–2·52). We found no trials assessing the effect of interventions on viral load outcomes in adolescents in LMICs; the studies that were reported from high-income settings were mainly cohort studies or analysed routinely collected programme data with inadequate control groups, had small sample sizes, and investigated interventions that are not feasible to implement in resource-limited settings.

**Added value of the study**

Our findings show that community-based support provided by community health workers to caregivers substantially reduces the risk of virological failure in HIV-infected children and adolescents. This is the first randomised trial of an intervention to improve treatment outcomes in children and adolescents, done in a LMIC, and with the outcome assessed using HIV viral load. The need for consent is often a major obstacle to including minors in studies and is one reason why few studies have been done in this age-group. This study adds to the sparse evidence base for interventions in older children and adolescents, in whom care outcomes have consistently been reported to be worse than in adults, and is also the first trial to show the effect of a community-based intervention on virological outcomes. The intervention focused on the caregivers who are key to children accessing care, and the trial showed an impact across the HIV care cascade.

**Implications of the available evidence**

In recent years, community-based interventions and use of community health workers have been widely promoted and recommended to relieve pressure on weak health systems and provide more patient-centred services. The study was done in a routine health-care setting using a cadre of workers that already exist in many African health systems, and therefore the intervention has high potential for scalability. Further studies that replicate this model are needed to understand contexual factors that will influence implementation and cost-effectiveness.

Caregivers are key mediators for children accessing and maintaining treatment, and their own physical and emotional health, understanding of HIV care, and access to supportive social networks affects their ability to ensure children's retention in care and adherence to treatment.^11^, ^12^ In two studies,^13^, ^14^ absence of a caregiver at a child's clinic appointments was associated with lower adherence to treatment and higher odds of virological failure. Formative research suggested that support for caregivers would lead to better outcomes in children living with HIV.^15^ We aimed to assess the effect of community-based support provided by trained community health workers to caregivers of children and adolescents living with HIV, on adherence to treatment and retention in HIV care in Harare, Zimbabwe.

## Methods

### Study design

In this open-label, randomised controlled trial, we recruited boys and girls aged 6–15 years with newly-diagnosed HIV attending seven primary health-care clinics in Harare, Zimbabwe. Ethical approval was granted by the Medical Research Council of Zimbabwe and the ethics committees of the Biomedical Research and Training Institute and the London School of Hygiene & Tropical Medicine, London, UK. Written informed consent from guardians and age-appropriate assent from participants were obtained.

### Participants

The study was done in seven communities in south-western Harare. Each community is served by a primary health-care clinic providing acute and antenatal care services. Provider-initiated HIV testing and counselling at all health facilities has been standard of care in Zimbabwe since 2007. The inclusion criteria were age 6–15 years, newly-diagnosed HIV infection, and residence in and planning to receive HIV care in one of the study communities.

Before the study, children were receiving HIV care including initiation of ART at hospital HIV outpatient services. This was in contrast to adults, of whom an increasing number were being initiated on ART at their nearest primary health-care clinic or transferred there for continuing care once they were stable on treatment. In a cross-sectional study in a public-sector HIV treatment clinic based at the largest hospital in Harare, of the children who had started ART between 2004 to 2011 aged 5–9 years and 10–14 years and had been taking ART for at least 6 months, the proportion who had a HIV viral load higher than 1000 copies/mL was 21% and 40%, respectively.^16^

From January, 2013, as part of the study, specialist nurse-led HIV care services for children were established at the seven study primary health-care clinics. Designated nurses from each primary health-care clinic and research nurses underwent training on delivery of paediatric HIV care including initiation of ART, at first-level facilities. The training used Ministry of Health and Child Care training tools for paediatric HIV care provision and the WHO Integrated Management of Childhood Illness clinical guidelines, and incorporated additional training on adolescent HIV care issues.

HIV treatment followed national guidelines and was provided to participants at primary health-care clinics by research nurses and routine clinical staff, supported by the study physician. This included cotrimoxazole prophylaxis for all patients, ART prescription (including ART initiation), management of intercurrent illness with referral to secondary care if indicated, and disclosure and adherence counselling. HIV treatment followed national guidelines (threshold of CD4 count <350 cells per μl until March, 2014; <500 cells per μl subsequently) and ART was provided by the National ART Programme. Those starting ART were seen once every 2 weeks for the first month, once a month for the next 2 months, and once every 3 months thereafter. Those not taking ART were followed up every 2 weeks for the first month and once every 3 months thereafter.

### Randomisation and masking

Children were randomly assigned (1:1) to receive decentralised primary health-care clinic-based HIV care (control group), monthly or every 3 months (according to national guidelines) for 18 months, or decentralised care plus structured support visits (either at home or at a location of the caregiver's and participant's choice) by trained community health workers (intervention group) according to national guidelines for 18 months. The target sample size for each clinic was proportional to the estimated number of children living with HIV, aged 6–15 years, in the clinic catchment area (determined through a previous enumeration survey).^17^

After consent to participate was obtained, a study number was allocated sequentially to each participant. Randomisation was done via random-number tables generated by a computer software programme by a statistician who had no role in the rest of the study; the data manager had sole access to the password-protected randomisation file. To ensure allocation concealment, the group allocation for each study number was provided through a mobile SMS to the recruiting research nurse. In households where more than one child was eligible, the first presenting child was enrolled, and subsequent children were allocated to the same group and were excluded from the trial analysis. Community health workers and nurses were not masked to allocation, but laboratory staff were. Masking was achieved by use of study ID numbers on forms.

### Procedures

At enrolment, the research nurses administered a clinical and social history to the caregiver using a structured questionnaire, and conducted a standardised examination, a brief HIV symptom screen was done, and incident clinical events (eg, admittance to hospital, unscheduled clinic visits, and infections), and height and weight were recorded and a prescription for cotrimoxazole and ART (where indicated) was given. The dose of ART was checked at every visit and adjusted according to weight. Self-reported adherence (reported by the caregiver for younger participants) was assessed once every 6 months using a visual analogue scale (VAS) and missed pills on 3-days recall.^18^ Participants who wished to transfer to another facility for care were given a referral and clinical history record and were not followed-up further.

HIV-1 viral load testing was done 12 months after ART initiation and 18 months after enrolment among those who initiated ART using the COBAS Ampliprep/Taqman 48 v2.0 platform (Roche Molecular Systems, Inc., Branchburg, NJ, USA). Participants who missed appointments were offered an alternative time, but were considered to have missed a visit even if they attended the alternative appointment. Participants were followed up for 18 months after randomisation, and continued to receive care at their primary health-care clinic following the end of the trial. Participants who were lost-to-follow-up were contacted via phone and home visits to ascertain their outcome.

The intervention used a strength-based case management approach, adapted from the Antiretroviral Treatment Access Study interventions.^19^ This approach focuses on skills, resources, and positive experiences in the face of adversity, and leads participants through a process of identifying barriers and practical solutions. Formative research, including a review of existing support programmes, mapping of local services, and qualitative interviews with caregivers, children and health-care providers, was undertaken to tailor the intervention to the local context.^15^

The intervention involved one-to-one sessions with children's primary caregivers delivered by community health workers, done over 18 months after enrolment at crucial points in a participant's progression through HIV diagnosis, treatment initiation, and long-term care, at a location of the caregiver's choice. The sessions were directed at the primary caregiver, although if the caregiver was not present, the community health worker could meet with another household member, relative or, rarely, the enrolled child, depending on age and family circumstances. The community health worker visits followed the clinic appointment schedule where possible. After two initial visits to establish rapport, there were three intensive and structured community health worker sessions and then shorter, less formal visits unless concerns were identified, in which case additional visits were scheduled. Both sessions aimed to assess the child's personal and family circumstances, support engagement with care, offer treatment literacy, clarify issues raised at clinic appointments, refer participants or families to local organisations offering additional support services, and provide a link to clinics by reminding caregivers of scheduled appointments. Clinic staff and community health workers met regularly to check participants' attendance and share issues that could be addressed within the clinic or community settings. The structure, timing, and content of the sessions are detailed in the appendix. Children not eligible for ART received the same community health worker visits to encourage retention in care during the pre-ART phase. Once they become eligible to initiate ART, the three intensive sessions were repeated to address treatment.

Eligibility criteria for community health workers included residence in the local community for at least 5 years, functional literacy (able to read and fill in rudimentary record-keeping forms, basic comprehension of literature such as health pamphlets), willingness to travel on foot between households and visit the local clinic, and experience of nurturing others (including caring for their own or others' children, sick or elderly family members, or previous work experience in a caring capacity). Community health workers underwent a 2 week training programme, followed by a further 2 weeks of intensive on-the-job supervision. The training programme had three components namely provision of HIV literacy (information related to HIV transmission, prevention and treatment), orientation to challenges faced by children and families in initiating and maintaining treatment, and instruction on intervention content and development of specific skills to achieve the objective of each visit. Training involved illustration through examples and case studies, role plays, and participatory activities (training resources available on request). During the intensive 2 week supervision period, each community health worker was shadowed for a day as they did visits followed by a debrief discussion. A refresher workshop was held at 2 months and then every 6–9 months. In addition, monthly meetings with the community health workers were held to discuss concerns, share initial experiences and problems, and facilitate a team approach to seek solutions to challenges confronted in their work. Community health workers were contracted on a probationary period for 2 months and their contract confirmed after a refresher workshop held at the end of the probationary period. Each community health worker was given a monthly stipend of US$20.

### Outcomes

The primary outcomes were the proportion of participants who had died or were virally unsuppressed (as defined by HIV-1 viral load ≥400 copies per mL) 12 months after starting ART, among those who initiated ART within 6 months of randomisation, and the proportion of all participants who missed two or more appointments in 18 months. A missed appointment was defined as failure to attend within 7 days after a scheduled appointment. This outcome was chosen as late visits result in exhaustion of drug supplies and missed doses. Findings of studies have consistently shown that missed appointments are associated with worse health outcomes.^20^ Those who transferred to another clinic within a month of enrolment (but not those who were lost to follow-up) were excluded from the analysis of missed visits because during their short follow-up they had fewer than two visits scheduled so they were not at risk of the outcome (missing two or more appointments).

A secondary, composite, outcome was the proportion of all participants who were virally unsuppressed, did not start ART, died, or were lost to follow-up, defined as the participant making no contact with the clinic for 6 months and not re-entering care elsewhere 18 months after enrolment. This outcome was selected to investigate the combined effect of the intervention on ART initiation, retention in care, and adherence. Participants who transferred to another clinic were excluded from analysis. Other secondary outcomes were all-cause mortality, number of hospital admissions (defined as stay in hospital for ≥24 h), number of unscheduled visits to a primary health-care clinic (defined as any clinic visit not pre-booked), all measured over 18 months; and caregiver or self-reported adherence (average adherence <90% over 12 months post-ART initiation; proportion who reported any missed pills in 3 days recall).

### Statistical analysis

The primary analyses were complete-case, modified-intention-to-treat. For binary outcomes, the odds ratio (OR) for the intervention effect was estimated using mixed-effects logistic regression, adjusting for clinic as a fixed effect and with random effects to account for correlation at the level of the community health worker. As specified a priori, analyses were adjusted for variables showing imbalance between groups at baseline. In addition, to produce an unbiased complete case analysis, analyses of primary outcomes and the composite secondary outcome were adjusted for variables associated with either the outcome or missingness. Hospital admission and unscheduled appointments were analysed as count outcomes using mixed-effects. Poisson regression, adjusted for unbalanced baseline variables and clinic as fixed effects, with community health worker as a random effect. Sensitivity analyses were performed; first, multiple imputation (20 imputations) using variables associated with the outcome and with outcome missingness for the first primary outcome and the composite secondary outcome; second, assuming either 0% or 100% of all participants who were in care at another clinic at follow-up were virally suppressed.

A sample size of 250 participants per group provides 80% power to detect a 40% reduction in the proportion of children missing two or more routine appointments, assuming a proportion of 30% in the control group and 30% loss-to-follow-up. Assuming 70% of children initiated ART within the first 6 months and with a year of follow-up, we expected 175 children per group to be eligible for the viral load outcome, giving 81% power to detect a 30% reduction in treatment failure/death from the expected 50% in the control group. Analyses were done with STATA v14.0 software (StatCorp, TX, USA).

The trial is registered with the Pan African Clinical Trials Registry, number PACTR201212000442288.

### Role of the funding source

The funder had no role in any aspect of study design or analysis, data collection, data analysis, data interpretation, writing of the report, or the decision to submit for publication. RAF, HW, TB, and VS had full access to all data. RAF had final responsibility for the decision to submit for publication.

## Results

**Figure 1:**
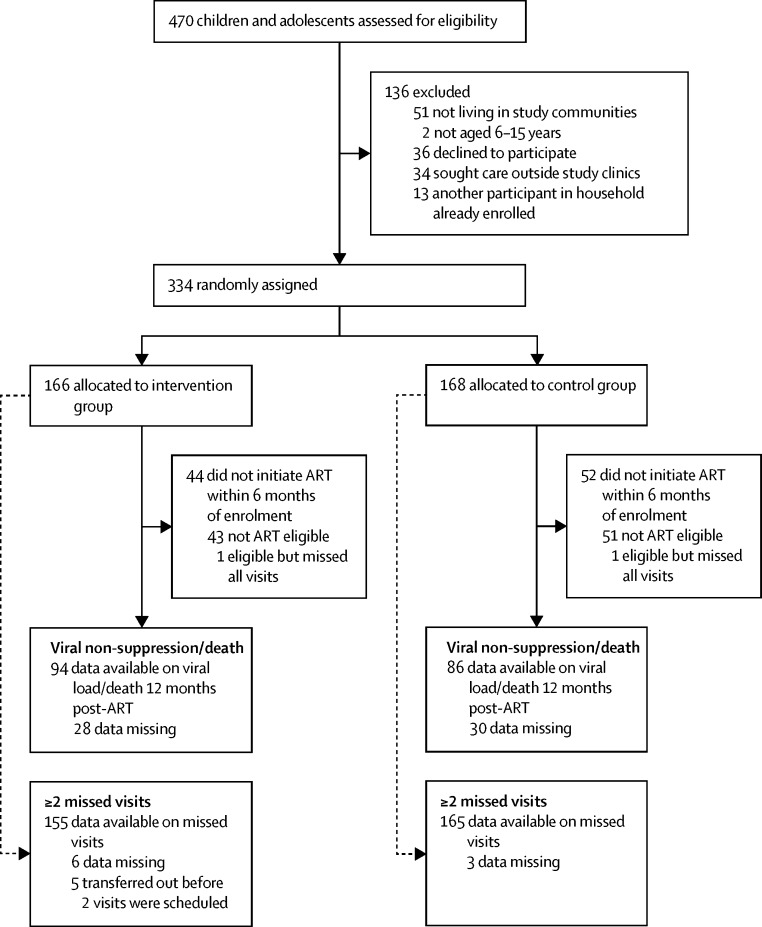
Trial profile

Between January, 2013, and January, 2015, 470 participants tested HIV-positive at study primary health-care clinics and were screened for eligibility. Of the 334 eligible children, 166 were randomly assigned to the intervention group and 168 to the control group (figure 1). The median age was 11 years (IQR 8–13 years), and 178 (53%) participants were girls (table 1). Because of slight imbalances in age and sex by group, these variables were adjusted for in outcome analyses. One participant withdrew from the trial 240 days after enrolment and 12 died during follow-up (five in the intervention group; seven in the control group). Of the remaining participants, 36 (22%) in the intervention group and 34 (20%) in the control group did not complete 18 months of trial follow-up, of whom 28 (17%) and 14 (8%) participants, respectively, had a facilitated transfer to another clinic with referral letters including a detailed clinical history to ensure continuity of care (table 2). There was no significant difference in the proportion of participants who missed two or more scheduled clinic visits (17% *vs* 18%; adjusted OR (aOR) 0·92, 95% CI 0·49–1·74; p=0·79; table 3). This proportion varied considerably by clinic, ranging from 2–44% (data not shown).

**Table 1:** Characteristics of participants at baseline and of the 238 participants who started ART within 6 months of enrolment

		**All trial participants**		**Participants who started ART within 6 months of enrolment**	
		Intervention group (N=166)	Control group (N=168)	Intervention group (N=122)	Control group (N=116)
Girls		91 (55%)	87 (52%)	70 (57%)	59 (51%)
Boys		75 (45%)	81 (48%)	52 (43%)	57 (49%)
Age (years)		11 (8–13)	11 (8–13)	11 (9–13)	11 (9–13)
Age group (years)					
	6–9	55 (33%)	62 (37%)	34 (28%)	32 (28%)
	10–12	61 (37%)	52 (31%)	46 (38%)	36 (31%)
	13–15	50 (30%)	54 (32%)	42 (34%)	48 (41%)
≥1 change in caregiver		102 (63%)	89 (54%)	79 (65%)	60 (52%)
Type of caregiver					
	Parent/step-parent	88 (53%)	95 (57%)	64 (53%)	64 (55%)
	Aunt/uncle	40 (24%)	32 (19%)	28 (23%)	21 (18%)
	Grandparent	27 (16%)	29 (17%)	22 (18%)	22 (19%)
	Sibling	9 (5%)	8 (5%)	6 (5%)	6 (5%)
	Nephew/niece	1 (1%)	2 (1%)	1 (1%)	1 (1%)
	Institution	1 (1%)	2 (1%)	1 (1%)	2 (2%)
Orphanhood status					
	Both parents alive	60 (36%)	71 (42%)	40 (33%)	46 (40%)
	Mother alive, father dead/unknown	37 (22%)	29 (17%)	28 (23%)	25 (22%)
	Father alive, mother dead/unknown	36 (22%)	35 (21%)	28 (23%)	21 (18%)
	Both parents dead/unknown	33 (20%)	33 (20%)	26 (21%)	24 (21%)
Health status					
	Wasting (weight for age z-score <–2)	32 (19%)	40 (24%)	29 (24%)	35 (30%)
	Stunting (height-for-age z-score <–2)	23 (14%)	30 (18%)	23 (19%)	25 (22%)
CD4 count (cells per μl)		378 (215–559)	376 (223–610)	286 176–463)	292 (160–413)
CD4 count ≤350 cells per μl		75 (45%)	78 (47%)	75 (62%)	73 (64%)

Data are n (%) or median (IQR).

**Table 2:** Retention in care at 18 months

	**Intervention group (N=166)**	**Control group (N=168)**
In care at the study clinic	125 (75%)	126 (75%)
Died	5 (3%)	7 (4%)
Withdrew from trial—but stayed in care at the same clinic	0	1 (1%)
Transferred to another clinic (planned transfer)	28 (17%)	14 (8%)
Transferred to another clinic without official transfer*	2 (1%)	9 (5%)
Moved away without forwarding address*	2 (1%)	5 (3%)
Untraceable*	4 (2%)	6 (4%)

* Ascertained through phone calls and home visits.

**Table 3:** Primary and secondary outcomes

		**Intervention group**	**Control group**	**Adjusted odds ratio (95% CI)**
**Primary outcomes**				
Proportion of patients who died or had an HIV viral load ≥400 copies per mL 12 months after ART initiation*		31/94 (33%)	42/86 (49%)	0·46 (0·23–0·89); p=0·02
Proportion of patients with ≥2 missing or late scheduled visits in 18 months†‡		27/155 (17%)	30/165 (18%)	0·92 (0·49–1·74); p=0·79
**Secondary outcomes**				
Composite outcome: proportion not initiating ART, virally unsuppressed, died or lost to follow-up 18 months after enrolment§		49/112 (44%)	69/119 (58%)	0·50 (0·28–0·89); p=0·02
All-cause mortality*		5/166 (3%)	7/168 (4%)	0·72 (0·22–2·41); p=0·59
Proportion with adherence <90% 12 months after ART initiation¶		7/94 (8%)	7/80 (9%)	0·75 (0·24–2·35); p=0·62
Proportion who missed all pills for ≥1 day out of the 3 previous days¶		3/94 (3%)	5/80 (6%)	0·58 (0·13–2·60); p=0·48
Number of hospital admissions†				
	0	154 (93%)	148 (88%)	0·59|| (0·31–1·12); p=0·11
	1	9 (5%)	15 (9%)	··
	2	3 (2%)	3 (2%)	··
	3	0	2 (1%)	··
Number of unscheduled attendances†				
	0	117 (71%)	121 (72%)	0·83|| (0·60–1·17); p=0·29
	1	40 (24%)	32 (19%)	··
	2	6 (4%)	8 (5%)	··
	3	2 (1%)	3 (2%)	··
	4–6	1 (1%)	4 (2%)	··

* 58 missing viral load: 29=non-attendance in the 40–75 week period; 5=viral load test failed; 24=child refused or logistic issues with sampling.

† Adjusted for clinic, age, sex, and community health worker (a priori).

‡ 14 missing outcome: 9=missing data; 5=transferred out within 30 days of enrolment.

§ Adjusted for clinic, age, sex, community health worker (a priori), baseline CD4 count (associated with outcome), wasting and stunting (associated with missingness). 103 missing outcome: 1=withdrew from trial; 53=transferred to another clinic for care; 49=logistic issues with blood sampling.

¶ Measured 41–75 weeks after ART initiation in 174 of 238 participants who started ART within 6 months; 9=died; 26=did not complete adherence questionnaire; 29=did not attend clinic during this period. If adherence measured more than once, closest time-point to a year post-ART initiation used for the outcome. Adjusted for age, sex, and community health worker.

|| Adjusted incidence rate ratio.

**Figure 2:**
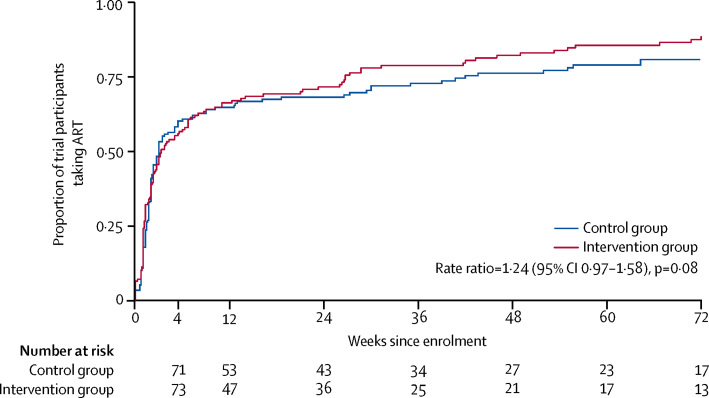
Proportion of participants initiated on ART over time, by trial group

6 months after enrolment, 238 (71%) participants had initiated ART, 122 (73%) in the intervention and 116 (69%) in the control group (table 1). At end of follow-up, 14% of participants still in care had not initiated ART, as they were still not eligible. The estimated rate ratio of initiating ART in the intervention compared to the control group was 1·24 (95% CI 0·97–1·58; p=0·08; figure 2). Among the 238 participants who initiated ART within 6 months of randomisation, 172 (72%) were followed up to 12 months after initiation and had a viral load measurement, while eight died within 12 months. The proportion with unsuppressed viral load or death was lower in the intervention group than the control group (31 [33%] of 94 participants *vs* 42 [49%] of 86, respectively; aOR 0·46, 95% CI 0·23–0·89; p=0·02; table 3). Among all participants, the proportion with unsuppressed viral load, death, not initiating ART, or who were lost to follow-up at 18 months was lower in the intervention group than the control group (49 [44%] of 112 participants *vs* 69 [58%] of 119 participants, respectively; aOR=0·50, 95% CI 0·28–0·89, p=0·02; table 3). Estimates of the intervention effect were similar using multiple imputation and sensitivity analyses for the composite outcome also showed similar results (appendix). In addition, in a post-hoc analysis, similar results were obtained for a composite endpoint of death, lost to follow-up, and unsuppressed viral load (38 [38%] of 101 in the intervention group *vs* 50 [50%] of 100 in the control group, aOR 0·53, 95% CI 0·28–0·99; p=0·046).

There was no evidence of an intervention effect on mortality, number of hospital admissions, or number of unscheduled clinic visits (table 3). No participant switched to second line treatment.

## Discussion

This is the first trial of an adherence support intervention to show an effect on HIV viral suppression in older children and adolescents. Our trial found 54% lower odds of virological failure or death among participants receiving community-based support by community health workers compared with those receiving HIV care solely at primary care facilities. Only half the participants achieved viral suppression in the control group (compared with two-thirds of those in the intervention group), despite provision of decentralised care provided by trained specialist nurses in both groups. This finding underscores the need for actively supporting adherence to treatment. Notably, although we noted a substantial intervention effect on viral suppression, there was no difference in self-reported adherence and participants in both groups probably over-estimated adherence.^21^ This has been reported by several studies and highlights the importance of using viral load measures to ascertain treatment outcomes.^22^, ^23^

There have been no adherence interventions that have improved virological outcomes among populations living with HIV in low and middle-income countries (LMIC), but trials have been sparse. In a network meta-analysis of randomised controlled trials aimed at improving adherence, cognitive behaviour therapy and supporter interventions had modest effects on HIV viral suppression compared with standard of care in all study settings, but none of the interventions improved viral load in studies done in LMICs.^24^ Support interventions including peer or community support have shown a modest effect on adherence but not on viral suppression in adults in LMICs, despite these interventions being widely promoted and recommended.^25^, ^26^ Most have been in high-income countries with interventions that are difficult to implement in resource-limited settings. In addition, the studies have been limited by small sample sizes, short follow-ups, lack of adequate control groups, and reliance on self-reported measures of adherence.^5^, ^6^

Although there was an effect of the intervention on viral load, there was no difference between groups in the proportion of participants who missed two or more clinic visits. This finding might be explained by the close proximity to the clinics and high quality of care provided by the research nurses in both groups. Notably, the proportion of missed visits varied substantially between clinics, suggesting that missing visits was affected by local context. For example, visits were more frequent than stipulated by national guidelines as adequate ART supplies to cover 3 months were not always available. Additional visits to collect prescriptions (counted as routine appointments) had to be scheduled and therefore the frequency of pharmacy visits varied by clinic. Reassuringly, the all-cause mortality and hospitalisation rates were low, which shows that it is feasible to provide effective paediatric HIV care in primary care settings.

By 18 months post-enrolment, 75% of participants were still engaged with care, with no difference by group. This finding is similar to findings from other pediatric cohorts in LMICs, which were mostly hospital-based.^27^ The intervention had an effect on the composite secondary outcome, which takes into account all the steps of the HIV care cascade following diagnosis namely assessment for ART eligibility, ART initiation, and adherence. Notably, the intervention was provided to all participants irrespective of whether they were eligible for ART. Community health workers were tasked to emphasise the importance of staying in care and to address participants concerns about their children starting ART. Despite trial groups having similar baseline CD4 counts, a higher proportion of intervention group participants were taking ART after 3 months of enrolment, and had significantly lower risk of the composite outcome (not starting ART, lost to follow-up, death, or virological failure) than the control group. Among those who left care at the study clinics, intervention group participants were significantly more likely to inform clinic staff of their intention to transfer to another clinic. This ensured a coordinated process for continuity of care, which is especially important for children who often experience unstable caregiving arrangements because of parental illness or orphanhood.^28^ Only 40% of participants had both parents alive and over half had had more than one change of caregiver.

The trial was done before the “treat all” strategy was recommended. However, most participants in our trial were eligible for ART at diagnosis and started ART shortly after diagnosis. With the treat-all strategy in this age group, a small proportion would not have been eligible based on national guidelines at the time of the study. We cannot extrapolate the effect of the intervention on viral load in this sub-group. Treating all irrespective of CD4 count does streamline the ART initiation procedure and it is possible that such an approach could reduce the difference in the composite outcome (which includes proportion who initiated ART). Notably, in a post-hoc analysis, there was still a significant difference in the composite outcome when ART initiation was excluded. Community support has been described as a safety net that improves adherence by reducing patients' social isolation, increasing their understanding of the importance of treatment, and helping to tackle stigma.^29^ Our trial was based on the hypothesis that HIV outcomes are affected by caregivers' awareness and willingness to invest effort into accessing care, and therefore supporting caregivers would translate into better outcomes for their children. The intervention mainly targeted caregivers, and improvement in viral suppression might have been mediated by caregivers' increased capacity to support children in taking treatment. Furthermore, community health workers were tasked with supporting caregivers to disclose to children their HIV status, which is associated with improved adherence and retention in care.^30^ A formal process assessment will be reported separately. Our intervention incorporated formalised management procedures, ongoing supervision and mentorship, monthly debriefs, and clear working guidelines. The intervention aligned with formal health system procedures, including regular meetings between community health workers and clinic staff. These components are crucial to the success and sustainability of community health worker programmes.^31^ It is important to note that while the intervention had a significant effect on viral suppression at 12 months after ART initiation and on the composite outcome, a substantial proportion in the intervention group still had poor outcomes. It is unlikely that one intervention will suffice to achieve optimum outcomes among children and adolescents; instead a multi-pronged approach will be required.

A major strength of this trial is that it was done in a routine health-care setting using a cadre of community health workers that exist in many African health systems, which means that implementing the intervention should be feasible in similar settings. However, context specific evaluations will be required to understand factors that would influence implementation such as cost and other local factors. Additionally, rigorous procedures were used to ascertain outcomes. Limitations include a smaller sample size than planned as HIV prevalence among older children attending primary health-care clinics was lower than in previous estimates, resulting in lower study power than originally planned. Nonetheless, there was strong evidence of an intervention effect. Viral load data were missing in a proportion of participants. Sensitivity analyses including multiple imputation showed similar effect estimates. Another limitation was a lack of cost-effectiveness data.

Health systems in many settings are overstretched as increasing numbers of individuals access ART. The 2016 WHO guidelines recommend treatment of all individuals living with HIV irrespective of disease stage, and a projected additional 21 million people globally will need ART.^32^ Furthermore, UNAIDS has set ambitious targets to identify 90% of HIV-positive persons through testing, to have 90% of diagnosed people on ART, and viral suppression in 90% of those on ART by 2020.^33^ Community based approaches to support retention in care and adherence are being promoted as a means to contribute to these targets.^32^ Our trial is the first to show the effectiveness of such an approach and the first adherence intervention to improve viral suppression in children and adolescents, an age group with disproportionately poor HIV treatment outcomes. To scale up community-based interventions, careful attention needs to be paid to training and mentoring community workers, addressing contextual issues, and monitoring for quality assurance.
