# Supplementary material for: The effect of community-based support for caregivers on the risk of virological failure in children and adolescents with HIV in Harare, Zimbabwe (ZENITH): an open-label, randomised controlled trial
Source: Lancet Child Adolesc Health. 2017 Nov;1(3):175–83. doi: 10.1016/S2352-4642(17)30051-2 (PMC5656092; doi:10.1016/S2352-4642(17)30051-2)
Supplement: Supplementary appendix [file mmc1.pdf]

THE LANCET
Child & Adolescent Health

Supplementary appendix

This appendix formed part of the original submission and has been peer reviewed. We post it as supplied by the authors.

Supplement to: Ferrand RA, Simms V, Dauya E, et al. The effect of community-based support for caregivers on the risk of virological failure in children and adolescents with HIV in Harare, Zimbabwe (ZENITH): an open-label, randomised controlled trial. *Lancet Child Adolesc Health* 2017; published online Sept 12. http://dx.doi.org/10.1016/S2352-4642(17)30051-2.

Supplement 1: Description of the ZENITH intervention

Aim of the intervention

Unlike adults, children are directly dependent on their caregivers for access to and engagement with healthcare, particularly at younger ages. Children's retention in HIV care and their adherence to treatment can rely on caregivers, extended family members, and support from the wider community, which all need to be targeted to optimise paediatric treatment success. The aim of this intervention is to improve outcomes in children by actively supporting caregivers to identify and address barriers to their child's engagement with HIV care.

HIV care cascade

Four distinct phases of treatment are commonly used in HIV programming, corresponding to points at which patients may require more intensive support to ensure optimal outcomes. These are (1) HIV Diagnosis, when patients require reassurance, information on next steps, and help accepting their status; (2) Antiretroviral therapy (ART) initiation, when they need to learn the requirements of their regimen and how to manage side effects; (3) Establishing long-term treatment maintenance, when the importance of adherence needs to be understood, as well as other aspects of living positively; and (4) Changes to treatment (or demonstrated poor adherence/appointment attendance), when patients may require help in re-committing to the treatment process and support to maintain adequate levels of motivation. Schedules of clinical appointments and counselling protocols often follow these four treatment stages, as do community-based outreach programmes.

Intervention content

The framework of the intervention is based on:

1) A family-based approach recognising that children's retention in care depends on not only on their own understanding and acceptance of their condition, but also on their specific family circumstances. These include, but are not limited to, relationship to a primary caregiver, HIV status of others living in the household, levels of open communication and support within and beyond the family, and access to resources (financial, instrumental and psychosocial). These

factors will likely change over time, particularly with increasing age of the child and at different stages of HIV treatment.

2) A strength-based case management approach, adapted from the Antiretroviral Treatment Access Study (ARTAS) interventions.[1-3] This focuses on individuals' skills, resources, and positive experiences in the face of adversity, rather than on constraints, and leads participants through a process of identifying potential barriers and practical solutions with support to achieve these solutions.

The intervention centres around a standardised set of support visits over an 18-month period, delivered by lay workers (LWs) at critical points in a child's progression through HIV diagnosis, treatment initiation, and long-term maintenance, including adherence to prescribed drug regimens. Facilitated discussions lead participants through a process of identifying their own strengths, planning for successful adherence, and developing practical solutions as challenges emerge. Referrals to available services in the area are offered, with practical assistance in following these up (such as contacting a local support group or checking eligibility for a food supplementation programme). Where possible, the visits are linked to clinical appointments, with regular meetings between nurses, counsellors, the project physician, and home visitors held to discuss patients and share information that may need to be addressed at either clinical monitoring appointments or within the community setting.

Timing & Content of Visits

The schedule of clinical care and HIV treatment follows national guidelines. The first 5-8 visits are structured around a series of standardised activities, followed by a further 7 less frequent visits that are shorter and less formal, unless particular concerns are identified (Figure 1).

Children who are eligible and choose to initiate ART receive counselling with the primary caregiver and start ART within 2 weeks. Subsequent clinical appointments are at 2 weeks, 6 weeks, and 10 weeks, after which 3 monthly appointments are given. Visits are integrated into this clinical schedule, with community-based follow-up occurring following a scheduled clinic appointment (Table 1).

Children who are not yet eligible to initiate ART are monitored at 3-monthly intervals. However, they will receive home visits in the same way as children on treatment, although each visit will be shorter and focus on the broader issues related to living with HIV, with only a brief overview of what they might eventually expect during treatment. Once the child initiates treatment, s/he receives the ART-focused structured activities (Table 2).

References

1. Craw JA, Gardner LI, Marks G, et al. Brief strengths-based case management promotes entry into HIV medical care: results of the antiretroviral treatment access study-II. J Acquir Immune Defic Syndr 2008;47:597-606.

2. Gardner LI, Metsch LR, Anderson-Mahoney P, et al. Efficacy of a brief case management intervention to link recently diagnosed HIV-infected persons to care. AIDS 2005;19:423-31.

3. Anti-Retroviral Treatment and Access to Services (ARTAS). An individual-level, multi-session intervention for people who are recently diagnosed with HIV: Implementation Manual. Washington, USA: Center on AIDS & Community Health (COACH).

Figure 1: Schedule of Clinic Follow-up

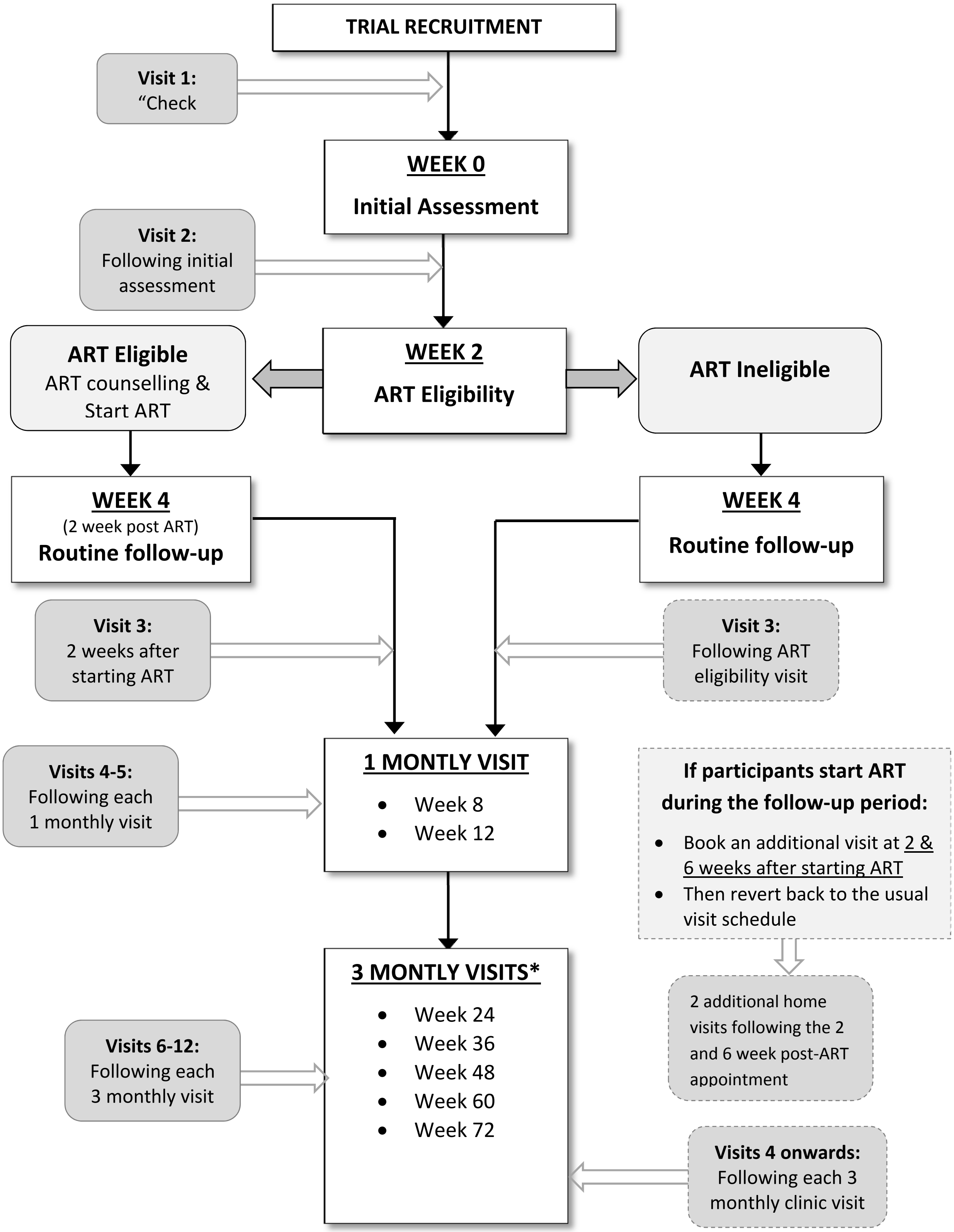

Table 1: Schedule and content of lay worker visits for participants eligible for ART at enrolment

Visit Number	When?	Content
**1. Check visit**	Between enrolment and Initial assessment	• Confirm address<br>• Introduce yourself to client and caregiver<br>• Introduction to home visits
**2. Introduction**	Within the 1st two weeks of ART initiation OR within a month of testing HIV+ (for those not yet eligible for treatment)	• Introduction to Home Visits<br>• Answering questions from clinical appointments regarding HIV, treatment, and ongoing monitoring<br>• Provision of information on HIV and treatment, including resources tailored to the age group of the child<br>• Family Mapping – activity to identify primary and secondary caregivers, their relationship to the child, and whether any household members are already on ART<br>• Identification of strengths and resources available to the caregiver, child, and household<br>• Assessing whether disclosure has been made to the child or other family/community members<br>• Assessing need for other family members to be tested
**3. Planning for successful treatment**	Within the next 2 weeks, following the first treatment monitoring clinic appointment for those who have	• Discussion of treatment experience to date<br>• Development of a Personal Treatment Plan by the caregiver (and/or child, depending on age and development), drawing on strengths and resources previously identified<br>• Assessment of need/eligibility for referrals to locally

	initiated ART	available services, support groups, etc. and provision of information on organisations active in the area<br>• Answering general questions as well as specific ones emerging from clinical appointments<br>• Discussion of how to manage drug stock-outs or additional treatment charges<br>• Follow-up on any changes in disclosure to child/others<br>• Follow-up on testing uptake by other household members
**4. Side Effects**	One month later (within 1 week of the next clinical appointment for those on ART, to follow-up any issues identified by clinic staff)	• Review of the Personal Treatment Plan, with discussion for how well it works and what changes are required<br>• Follow-up on referrals made at previous visit, and whether these have been taken up, need assistance, or new referrals are required<br>• Facilitated discussion around side effects, experience to date, and how to manage them<br>• Provision of information on managing side effects, including age-appropriate factsheets<br>• Answering general questions as well as specific ones emerging from clinical appointments<br>• Follow-up on any changes in disclosure to child/others<br>• Follow-up on testing uptake by other household members
**5. Disclosure**	One month later,	• Review of the Personal Treatment Plan, with

	after 2nd monthly follow-up appointment for those on treatment, and after 1st 3-monthly assessment for those not yet eligible<br>*NB: Once child becomes eligible, Visits 1-3 are repeated, with focus on ART-specific activities*	discussion for how well it works and what changes are required<br>• Facilitated discussion around disclosure to the child, with suggestions (and provision of materials) for how to disclose to children at different ages; offer to provide assistance with disclosure at a subsequent visit<br>• Answering general questions as well as specific ones emerging from clinical appointments<br>• Follow-up on referrals<br>• Follow-up on testing uptake by other household members
**6: Maintenance**	One month later	• Review of the Personal Treatment Plan, with discussion for how well it works and what changes are required<br>• Check on disclosure to the child (reactions/emerging issues if child aware of status; offer to assist with disclosure if child not yet informed)<br>• Facilitated discussion around long-term maintenance of treatment, including ongoing commitment and reminder of identified strengths & resources that will support the treatment plan<br>• Answering general questions as well as specific ones emerging from clinical appointments<br>• Follow-up on referrals

		• Follow-up on testing uptake by other household members
**7-12: Ongoing Support**	Every 3 months	• Follow-up on issues emerging from clinical monitoring appointments<br>• Review of Personal Treatment Plan and experience with treatment<br>• Review of need for referrals, assistance with disclosure, and/or support for testing of other household members<br>• Answering questions, facilitating identification of solutions to emerging challenges, and providing relevant information

Table 2: Schedule and content of lay worker visits for participants ineligible for ART at enrolment

Visit Number	When?	Content
**1. Check visit**	Between enrolment and Initial assessment	• Confirm address<br>• Introduce yourself to client and caregiver<br>• Introduction to home visits
**2: Introduction**	Within a week of initial assessment	• Answering questions from clinical appointments regarding HIV, treatment, and ongoing monitoring<br>• Provision of information on HIV and treatment, including resources tailored to the age group of the child.<br>• Family Mapping – activity to identify primary and secondary caregivers, their relationship to the child, and whether any household members are HIV+ve /already on ART<br>• Identification of strengths and resources available to the caregiver, child, and household<br>• Assessing whether disclosure has been made to the child or other family/community members<br>• Assessing need for other family members to be tested
**Home visits continue to follow the schedule of clinic visits.<br>Once a child becomes eligible for ART, conduct the 3 visits focused on treatment and adherence**		
**3: Disclosure**	One month later, after 1st follow-up appointment (Week 4)	• Conversation about recent monitoring appointment, including the significance of being found to be ineligible for ART.<br>• Emphasis on positive living, to remain healthy without treatment.<br>• Answering questions emerging from recent appointment or general

		• Reinforcing importance of maintaining monitoring appointments to check for eligibility and manage other health issues.<br>• If child does not know status: facilitated discussion around disclosure, with suggestions (and provision of materials) for how to disclose to children at different ages; offer to provide assistance with disclosure at a subsequent visit<br>• Assessment of need/eligibility for referrals to locally available services, support groups, etc. and provision of information on organisations active in the area<br>• Follow-up on testing uptake by other household members
**4. Ongoing Support**<br><br>***Until eligibility or end of follow up***	Every 3 months	• Follow-up on issues emerging from clinical monitoring appointments<br>• Review of need for referrals, assistance with disclosure, and/or support for testing of other household members<br>• Emphasis on positive living, to remain healthy without treatment.<br>• Answering questions emerging from recent appointment or general<br>• Reinforcing importance of maintaining monitoring appointments to check for eligibility and manage other health issues.
**Visits to be inserted if/when child initiates ART**		
**Planning for successful treatment**	Within 2 weeks of initiating ART	• Discussion of treatment experience to date<br>• Development of a Personal Treatment Plan by the caregiver (and/or child, depending on age and development), drawing on strengths and resources previously identified (may need to

		repeat the exercise, if visit 2 occurred a long time ago)<br>• Answering questions emerging from clinical appointments<br>• Discussion of how to manage drug stock-outs or additional treatment charges<br>• Follow-up on any changes in disclosure to child/others<br>• Follow-up on testing uptake by other household members<br>• Follow-up on referrals previously made/taken up
**Side Effects**	Within 1 week of the 1st monthly ART monitoring appointment	• Review of the Personal Treatment Plan, with discussion for how well it works and what changes are required<br>• Facilitated discussion around side effects, experience to date, and how to manage them<br>• Provision of information on managing side effects, including age-appropriate factsheets<br>• Answering general questions as well as specific ones emerging from clinical appointments<br>• Follow-up on any changes in disclosure to child/others<br>• Follow-up on testing uptake by other household members
**Maintenance**	Within 1 week of the 2nd monthly ART monitoring appointment	• Review of the Personal Treatment Plan, with discussion for how well it works and what changes are required<br>• Check on disclosure to the child (reactions/emerging issues if child aware of status; offer to assist with disclosure if child not yet informed)<br>• Facilitated discussion around long-term maintenance of treatment, including ongoing commitment and reminder of identified strengths & resources that will support the treatment plan<br>• Answering general questions as well as specific ones emerging

		from clinical appointments<br>• Follow-up on referrals<br>• Follow-up on testing uptake by other household members
